# Studies on Optimization of Growth Parameters for L-Asparaginase Production by *Streptomyces ginsengisoli*

**DOI:** 10.1155/2014/895167

**Published:** 2014-01-29

**Authors:** Neelima Deshpande, Prachi Choubey, Manasi Agashe

**Affiliations:** Department of Microbiology, Abasaheb Garware College, Pune 411004, India

## Abstract

A species of *Streptomyces*, *Streptomyces ginsengisoli*, a river isolate, was evaluated for production of an enzyme, L-asparaginase, with multiple functions mainly anticancer activity. The actinomycete was subjected to submerged fermentation by “shake flask” method. The quantity of L-asparaginase produced was estimated as 3.23 **μ**mol/mL/min. The effect of various culture conditions on L-asparaginase production was studied by adopting a method of variation in one factor at a time. Of the various conditions tested, glucose (followed by starch) and peptone served as good carbon and nitrogen sources, respectively, for maximal production of enzyme at pH 8. The temperature of 30°C and an incubation period of 5 days with 0.05 g% asparagine concentration were found to be optimum for L-asparaginase production.

## 1. Introduction

L-asparaginase (L-asparagine amido hydrolase E.C.3.5.1.1) enzyme which converts L-asparagine to L-aspartic acid and ammonia has been used as a chemotherapeutic agent in the treatment of acute lymphoblastic leukemia for over 30 years [1]. The clinical action of this enzyme is attributed to the reduction of L-asparagine, since tumor cells unable to synthesize this enzyme are selectively killed by L-asparagine deprivation [1–3].

Due to its prompt therapeutic potential, screening of microbial sources for asparaginase activity has been greatly intensified and well documented in filamentous fungi, yeast and bacteria like *E. coli *[4, 5], *Vibrio succinogenes* [6], *Erwinia carotovora* [7, 8], and *Bacillus *sp. [9, 10]. Among the fungi *Mucor *sp. [11], *Penicillium *sp. [12, 13], and yeast-like *Candida utilis* [14] have been proved to be potential producers of this enzyme.

Unfortunately the rapid development of side effects has limited the usage of currently available L-asparaginase. PEG-asparaginase, a form of *Escherichia coli *L-asparaginase covalently linked to polyethylene glycol, was rationally synthesized to decrease immunogenicity of the enzyme and prolong its half-life [1].

Actinomycetes are recognized as comparatively less explored source for L-asparaginase and therefore act as candidates for the production of L-asparaginase [15, 16]. Actinomycetes like *S*. *griseoluteus *[17], *S*. *karnatakensis *[18], *S*. *venezuelae *[19], *S. gulbargensis* [3], and *Nocardia levis* were reported to be potential candidates.

The present investigation deals with isolation and characterization of L-asparaginase from river sediment isolates of actinomycetes as a novel source of enzyme which is effective against leukemia. The isolate was identified as *Streptomyces ginsengisoli* by 16S r-DNA sequencing studies. Further analysis deals with optimization of different parameters for L asparaginase production.

## 2. Material and Methods

### 2.1. Isolation and Screening of Actinomycetes for Production of L-Asparginase by Plate Assay

The actinomycetes were isolated from river sediment samples collected from various locations in India. Seven isolates were screened for L-asparaginase production by plate assay [20]. The medium used for screening was Kuster's medium. The principle on which this screening was based is as follows: The medium contained phenol red indicator which turns pink in colour under alkaline conditions. The enzyme L-asparaginase will act on the substrate L-asparagine leading to production of ammonia which shifts the pH towards alkaline. The pink zone around the colony indicates that the organism is able to produce L-asparaginase. Thus this qualitative method was used as a screening test for L-asparaginase production.

### 2.2. Identification of Selected Isolate Was Carried out by 16s r-RNA Sequencing Followed by BLAST Analysis

#### 2.2.1. Cultivation of Actinomycetes

In order to obtain sufficient growth and maximum enzyme production from the identified strain, different media selected from the literature, that are used for cultivation of Actinomycetes were used [21–24] and the one giving maximum enzyme activity was used for further analysis.

### 2.3. Determination of Production Profile of L-Asparaginase

For determining the production profile of L-asparaginase, actinomycete colonies from the plate were directly inoculated into asparagine dextrose salt broth (ADS) containing 1% dextrose, 0.05% L-aspargine, 0.05% di potassium hydrogen phosphate, and 0.2% meat extract with initial pH 7. The inoculated flasks were then incubated at 30°C for 7 days in order to estimate the cell growth of the strain as well as L-asparaginase production for every 24 h interval. Cell growth was expressed in terms of dry weight of biomass (mg/mL) [18].

L-asparaginase activity was measured by following method [23, 25]. The cultures in ADS broth were centrifuged at 8000 rpm for 30 mins and the resultant supernatant, that is, the crude extract, was used to determine the L-asparaginase activity. Reaction was started by adding 0.5 mL of crude cell free extract into 0.5 mL of 0.04 M L-asparagine solution and 0.5 mL of 0.05 M Tris HCL buffer pH 8 and incubated at 37°C for 30 mins in water bath. The reaction was stopped by addition of 0.5 mL of 1.5 M TCA (trichloroacetic acid). Precipitated proteins were removed by centrifugation at 8000 rpm for 15 mins at 4°C, and the ammonia released was determined spectrometrically at 425 nm by nesslerization (by Nessler's method). Tubes kept at zero time incubation served as control. Enzyme activity was determined on the basis of liberation of ammonia calculated with reference to a standard curve of ammonium sulphate. In the concentration range of 0.1 mL of supernatant was added to 3.75 mL of distilled water, followed by addition of 0.2 mL of Nessler's reagent and was incubated at room temperature for 10 mins after which the absorbance was taken at 425 nm.

*Enzyme Unit.*One unit of asparaginase is the amount of enzyme which catalyzed the formation of 1 umol of ammonia per minute at 37°C [23].

The cell dry weight was recorded simultaneously at the interval of 24 hrs by drying the cell debris collected after centrifugation in an oven at 90°C for 24 h.

### 2.4. Effect of Sonication

100 mL of ADS broth was centrifuged at 10,000 rpm for 20 minutes followed by separation of cell pellets, to which 0.05 mM Tris buffer (pH 7.8) (5 mL) was added. Sonication was carried out with 30 pulses for 6 minutes at 4°C. The lysed cell pellet was then centrifuged at 47,000 g for 12 min and enzyme activity of the cell-free supernatant after sonication and that of a cell pellet after sonication were determined [26].

### 2.5. Optimization of L-Asparaginase Production (One Factor at a Time)

#### 2.5.1. Initial pH and Temperature Optimization

Impact of pH on the production of L-asparaginase was examined by culturing the strain in ADS broth adjusted to various pH levels ranging from 4.0 to 10.0. The optimal pH achieved at this step was used for further study. To determine the optimum temperature for L-asparaginase production, the strain was cultured in ADS broth at different temperatures ranging from 25 to 50°C for an incubation period of 5 days [2].

#### 2.5.2. Carbon Source

To investigate the effect of carbon sources on L-asparaginase production by the strain, ADS broth was supplemented with different carbon sources such as starch, mannitol, lactose, sucrose, and glucose each at a concentration of 1% (w/v) [3, 27] keeping other components constant.

#### 2.5.3. Nitrogen Source

Different nitrogen sources, namely, beef extract, yeast extract, and peptone, were added at a concentration of 0.2% (w/v) to ADS broth keeping other ingredients constant except meat extract [3]. Inorganic nitrogen source was not tried as it would interfere in enzyme assay.

#### 2.5.4. Effect of Aeration

To study the effect of aeration, two sets of flasks were incubated; one flask was incubated under stationary conditions and another was kept on shaker at 120 rpm for 5 days using ADS broth.

All the experiments were performed in triplicates and results were expressed as an average value with standard deviation.

## 3. Results and Discussion

### 3.1. Enrichment, Isolation, and Screening for L-Asparaginase Production by Plate Assay

**Figure 1:**
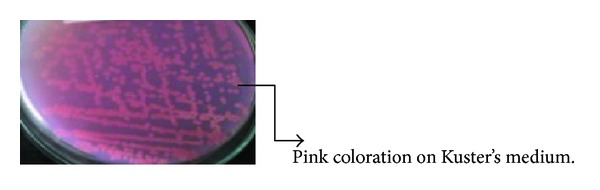
The results of plate assay method for qualitative determination of enzyme production by the isolate.

Isolates obtained were screened for the production of the enzyme L-asparaginase by using plate assay method (qualitative method). Results obtained showed higher intensity of pink coloration for three out of seven cultures tested (Figure 1).

Cultures giving positive test with plate assay method were then checked for enzyme activity quantitatively. Results obtained showed that one of the three tested cultures gave higher enzyme activity and therefore it was selected for further optimization studies on L-asparaginase production.

### 3.2. Identification of the Selected Strain of Actinomycetes

Identification of the selected strain was carried out by 16s r-RNA sequencing and the isolate was identified as *Streptomyces ginsengisoli*. (GenBank Accession number KF649337).

### 3.3. Use of Different Media for Growth and Production of Enzyme

Comparative analysis was carried out by inoculating the culture in different media in order to obtain increased growth and production of the enzyme. Results revealed that higher enzyme activity was observed in ADS broth as compared to other media used (Table 1). ADS broth was selected for all further experiments.

### 3.4. Production Profile of L-Asparaginase

**Figure 2:**
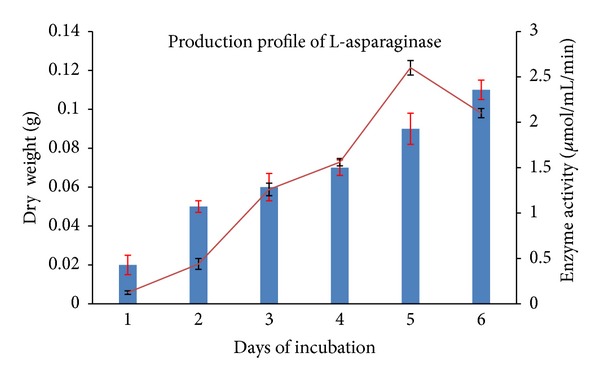
Production profile of L-asparaginase by *Streptomyces ginsengisoli* grown in asparagine dextrose salt Broth. A positive correlation between cell dry weight (bar chart) and enzyme activity (line) was observed.

Production of L-asparaginase by *Streptomyces ginsengisoli* started after 24–32 h of cell growth. Good correlation between cell growth and enzyme activity was observed when daywise analysis of dry weight and enzyme activity was carried out. Maximum activity was observed on the fifth day of incubation from the day of inoculation of the culture (Figure 2). A positive correlation between enzyme activity and cell mass over a time period of 24–120 hours has also been reported by Priya et al., in their work on *Streptomyces *sp. (TA22) isolated from Western Ghats, lndia [24].

There are few reports on actinomycetes producing predominantly extracellular L-asparaginase, while majority of bacterial species and few actinomycetes like *Streptomyces*. *karnatakensis *[18], and *Streptomyces albidoflavus *[27] are reported to be cell bound [1]. Comparison of extracellular and intracellular enzyme activity of *Streptomyces ginsengisoli* revealed that enzyme produced by the culture is predominantly extracellular. (Table 2).

### 3.5. Optimization of L-Asparaginase Production

#### 3.5.1. Initial pH and Temperature Optimization

**Figure 3:**
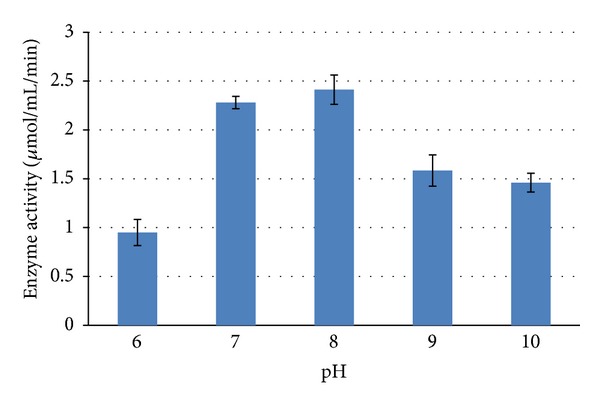
Effect of initial pH levels of the medium on production of L-asparaginase by *Streptomyces ginsengisoli. *

A study of initial pH levels (6–10) of ADS broth on production of L-asparaginase by *Streptomyces ginsengisoli* indicated an optimal enzyme activity at pH 8.0 (Figure 3). Optimum pH for production of L-Asparaginase by *Streptomyces albidoflavus* has been reported to be with the range of 7.5–8 which was found to be in accordance with our work [27].

**Figure 4:**
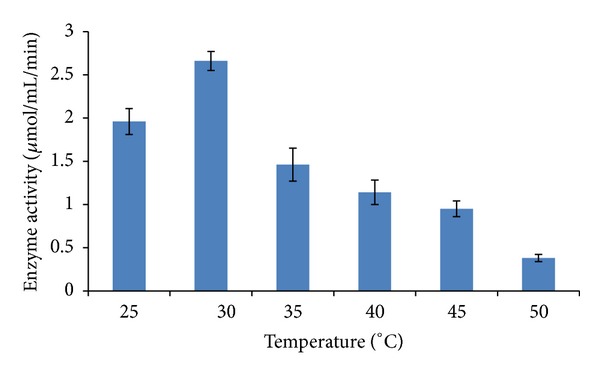
Effect of different levels of incubation temperature on production of L-asparaginase by *Streptomyces ginsengisoli*. Values are the means of three replicates ± SD.

Impact of temperature on L-asparaginase production was studied over a temperature range of 25°C to 50°C where maximum enzyme production was observed at 30°C (Figure 4). Actinomycetes like *Streptomyces albidoflavus* have been reported to produce L-asparaginase within the temperature range of 30–35 [6]. The present study revealed that L-asparaginase production was high when the culture was grown in ADS broth with an initial pH 8.0 and 30°C incubation.

#### 3.5.2. Carbon Sources

**Figure 5:**
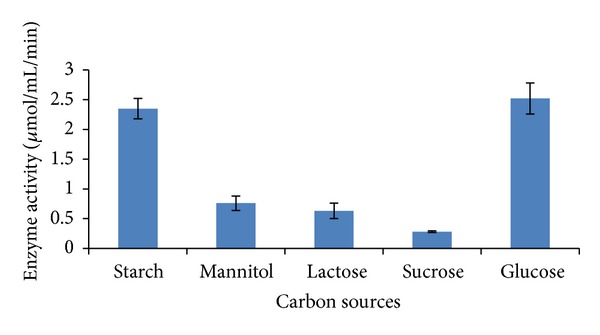
L-asparaginase production by *Streptomyces ginsengisoli* grown in asparagine dextrose salt broth amended with different carbon sources.

Different carbon sources like starch, mannitol, lactose, sucrose, and glucose were amended in asparagine dextrose salt broth to determine their impact on L-asparaginase production (Table 3) (Figure 5). As compared to other carbon sources tested, L-asparaginase production was high in presence of glucose. Comparable results were also obtained when starch was used as a carbon source. Maximum L-asparaginase production has been reported by *Amycolatopsis* CMU-H002 when ADS broth was amended with starch as the carbon source [23]. Biosynthesis of L-asparaginase by *Streptomyces albidoflavus* has been reported to be higher when the basal medium was supplemented with starch [27]. Sucrose, when added to the basal medium, served as a good carbon source for L-asparaginase production by actinomycetes isolated from estuarine fishes [25].

#### 3.5.3. Nitrogen Sources

**Figure 6:**
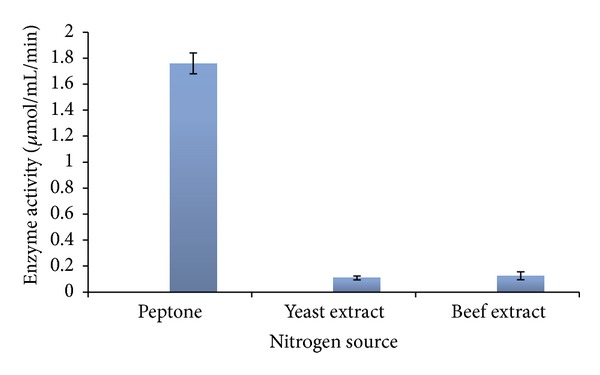
L-asparaginase production by *Streptomyces ginsengisoli* grown in asparagine dextrose salt broth amended with different nitrogen sources.

Effect of nitrogen compounds on production of L-asparaginase by *Streptomyces ginsengisoli* was studied by incorporating different nitrogen sources to ADS Broth containing 1% glucose. L-asparaginase production varied with the three nitrogen sources tested. Among them culture medium amended with peptone favored maximum enzyme production by this strain (Table 4) (Figure 6). For production of L-asparaginase by *Streptomyces albidoflavus*, yeast extract has been reported as a good nitrogen source [27]. Similar results have been reported by Khamna et al. in their studies associated with production of L-asparaginase from isolates obtained from rhizosphere soil of Thai medicinal plant [23].

#### 3.5.4. Effect of Aeration

**Figure 7:**
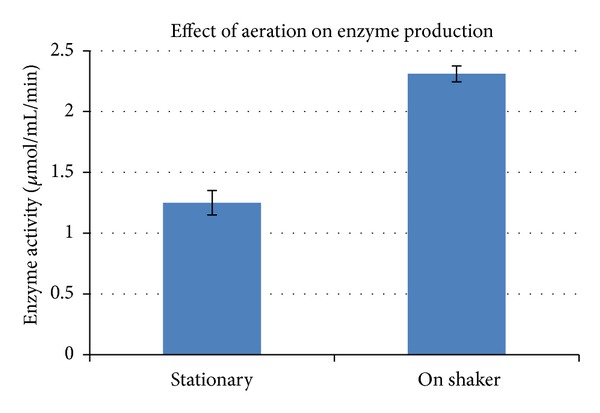
Effect of agitation on L-asparaginase production by *Streptomyces ginsengisoli.* Values are the means of three replicates ± SD.

Considerable decrease in enzyme activity was observed when culture was allowed to grow under stationary conditions as compared to the one kept on shaker at 120 rpm (Figure 7). Enzyme activity of the optimized media was found to be 28% more than the unoptimized media (Table 5). A correlation between agitation speed and enzyme production has still been reported by Jain et al. in their work on *Streptomyces gulbargensis* [16].

The present study revealed that all the selected parameters examined showed a considerable impact on L-asparaginase production by the novel isolate *Streptomyces ginsengisoli*. High activity of the enzyme was obtained at pH 8 and temperature 30°C. Maximum enzyme production was achieved when asparagine dextrose salt broth was supplemented with glucose and peptone as carbon and nitrogen sources, respectively. Optimization of growth parameters showed significant effect on production of enzyme L-asparaginase from 2.52 to 3.23 *μ*mol/mL/min. Thus, use of optimized asparagine dextrose salt broth increased enzyme activity by 28%. To the best of our knowledge this is the first report on production of L-asparaginase enzyme by *Streptomyces ginsengisoli*. The present work was aimed at evaluation of the parameters for its production and not at isolation and characterization of the enzyme. Moreover the work does not include investigation of the anticancer effect of L- Asparaginase.

## Figures and Tables

**Table 1:** L-asparaginase enzyme activity by *Streptomyces ginsengisoli* on different media.

Name of the medium	Enzyme activity (*μ*moles/mL/min)
Bennett's broth	1.04 ± 0.04
Yeast extract malt extract	0.92 ± 0.06
Asparagine dextrose salt broth (ADS)	2.53 ± 0.12
Bennett's broth + 0.05 L-asparagine	1.268 ± 0.07
Bennett's broth + 0.05 L-asparagine (casein hydrolysate replaced)	1.712 ± 0.09
Bennett's broth + 0.2 L-asparagine (casein hydrolysate replaced)	1.206 ± 0.25

Values are the mean of three replicates ± SD.

**Table 2:** 66% of the enzyme activity was found to be extracellular when compared with intracellular enzyme activity.

	*Streptomyces ginsengisoli *	Enzyme activity (*μ*mol/mL/min)	Enzyme activity (%)
Extracellular	Cell-free supernatant (before sonication)	2.412 ± 0.19	66
Intracellular	Supernatant (after sonication)	0.888 ± 0.03	25
	Precipitate (after sonication)	0.316 ± 0.12	9

For optimization of enzyme production, extracellular enzyme activity was considered.

**Table 3:** L-asparaginase production by *Streptomyces ginsengisoli* grown in asparagine dextrose salt broth amended with different carbon sources.

Carbon source	Enzyme activity (*μ*mol/mL/min)
Starch	2.348 ± 0.17
Mannitol	0.76 ± 0.12
Lactose	0.632 ± 0.13
Sucrose	0.28 ± 0.015
Glucose	2.52 ± 0.26

Values are the means of three replicates ± SD.

**Table 4:** L-asparaginase production by *Streptomyces ginsengisoli* grown in asparagine dextrose salt broth amended with different nitrogen sources.

Nitrogen source	Enzyme activity (*μ*mol/mL/min)
Peptone	1.76 ± 0.13
Beef extract	0.126 ± 0.15
Yeast extract	0.1904 ± 0.015

Values are the means of three replicates ± SD.

**Table 5:** Comparison of enzyme activity obtained before and after optimization where 28% higher activity was obtained for optimized asparagine dextrose salt broth.

	Before optimization	After optimization
Enzyme activity (*μ*mol/mL/min)	2.52	3.23
